# Syndrome of Inappropriate Antidiuretic Hormone in Esophageal Cancer Patient

**DOI:** 10.1155/2021/8131834

**Published:** 2021-01-19

**Authors:** Rahmawati Minhajat, Andi Fachruddin Benyamin, Andi Makbul Aman, Syakib Bakri

**Affiliations:** ^1^Division of Hematology and Medical Oncology, Internal Medicine Department, Medical Faculty Hasanuddin University, Makassar, Indonesia; ^2^Histology Department, Medical Faculty Hasanuddin University, Makassar, Indonesia; ^3^Division of Endocrinology and Metabolism, Internal Medicine Department, Medical Faculty Hasanuddin University, Makassar, Indonesia; ^4^Division of Nephrology and Hypertension, Internal Medicine Department, Medical Faculty Hasanuddin University, Makassar, Indonesia

## Abstract

Syndrome of inappropriate antidiuretic hormone (SIADH) is a disorder of fluid and sodium balance characterized by hypotonic hyponatremia, low plasma osmolality, and increased urine osmolality caused by excessive release of antidiuretic hormone (ADH). Malignancy is one of the most common causes of SIADH, but SIADH in esophageal carcinoma is very rarely reported. In this case report, a 74-year-old male patient of moderate differentiation of squamous cell esophageal carcinoma had a recurrent electrolyte balance disorder despite repeated corrections. The patient experienced improvement after fluid restriction and drug administration.

## 1. Introduction

Syndrome of inappropriate antidiuretic hormone (SIADH) or commonly called Schwartz-Bartter syndrome is a disorder of fluid and sodium balance characterized by hypotonic hyponatremia, low plasma osmolality, and increased urine osmolality caused by excessive release of antidiuretic hormone (ADH), whether from posterior pituitary or other sources, in the absence of kidney disease or other nonosmotic stimuli identified which can induce ADH release [1, 2].

Schwartz, in 1957, first reported the SIADH case in two lung cancer patients who had hyponatremia whose cause was unknown. The cause is thought to be due to the tumor producing substances similar to ADH [3]. Malignancy is one of the most common causes of SIADH, although many other nonneoplastic causes including lung disease, central nervous system (CNS) disorders, and the use of certain drugs associated with the occurrence of SIADH [2, 4, 5]. Reportedly, around 67% of SIADH cases were found in cancer patients, where the majority (70%) were type of small cell lung cancer. Very rare cases of SIADH are reported in patients with esophageal carcinoma and carcinoma in other tissues with histological features of squamous cell carcinoma [5]. Hyponatremia due to SIADH is associated with increased morbidity and mortality from the underlying disease [4, 6, 7].

The diagnosis of SIADH is based on Bartter and Schwartz criteria that consist of hyponatremia (serum sodium lower than 130 mEq/1), plasma osmolality lower than 275 mOsm/kg, urine osmolality higher than the plasma osmolality and above 500 mOsm/kg, absence of clinical evidence of volume depletion, normal renal and adrenal function, and normal thyroid function [3].

## 2. Case Report

A 74-year-old male was admitted to hospital with a chief complaint of general weakness since the last 3 months and increasingly burdensome. There was nausea, vomiting, but no heartburn, decreased appetite for the last 5 months, and weight loss of around 7 kg in the last 6 months. There is no fever, headaches are often felt since the last 6 months and usually do not improve even after taking analgetics, and patients also sometimes find it difficult to sleep. There is no cough and dyspnoea; the patient often feels pain in the right chest which only decreases after taking analgetics. Urination is smooth, but the volume of urine was less, irregular defecation as long as this complaint.

Previous medical history: the patient was treated at hospital with a diagnosis of moderate differentiation of invasive squamous cell esophageal carcinoma, suspected lung and brain metastases with electrolyte balance disorders. The patient received chemotherapy 13 times and radiotherapy 30 times during treatment at a hospital in another country six months ago. Laboratory results of the last serum sodium after chemotherapy is 124 mmol/L. One month before admission in our hospital, the patient received a total of 10 bottles of 3% sodium infusion in another hospital. The serum sodium levels after correction were 111 mmol/L, 112 mmol/L, and 117 mmol/L, respectively. There was no history of swelling, hypertension, diabetes mellitus, and thyroid disease.

Physical examination found vital signs within normal limits, good consciousness, and body mass index (BMI) 18.4 kg/m^2^. No signs of anemia and jaundice, no palpable tumor mass, and no lymphadenopathy were found. Thoracic, cardiac, and abdominal physical examinations were within normal limits. There was no extremity edema and abdominal ascites.

The results of laboratory tests are as follows: leukocytes 4,600/mm^3^, erythrocytes 3.35 × 10^6^/mm^3^, hemoglobin 10.6 gr/dL, hematocrit 28.5%, platelets 99,000/mm^3^, MCV 85.1 pg, MCHC 37.0 gr/dL, neutrophils 76.9%, lymphocytes 20.1%, RBG 156 mg/dL, ALT 17 U/L, AST 14 U/L, creatinine 0.9 mg/dL, blood urea 26 mg/dL, sodium 117 mmol/L, potassium 3.2 mmol/L, clorida 83 mmol/L, and plasma osmolality 216 mMol/kg. Other examinations found normal electrocardiography and abdominal ultrasound within normal limit, except for cysts in the right kidney (6.5 × 5.7 mm). Histopathological examination of esophageal tumor tissue biopsy was moderate differentiation of invasive squamous cell esophageal carcinoma. Thoracic CT-scan impression was sicculer esophageal tumors as high as the middle third with a mark of adhesions with the posterior portion of the aorta, no metastasis to lymph nodes and lung. The impression of PET-CT scan results postradiotherapy was increased FDG uptake is seen at a long segment of the thoracic esophagus. In view of previous radiation, considerations include esophagitis versus residual tumor. No increase was seen at the right hilum. The previously seen coeliac axis node is much smaller and non-FDG-avid. There is no evidence of hypermetabolic hepatic metastasis. A tiny nodule in the lingula is nonspecific. Increased uptake is seen at the right parietal brain parenchyma. This may be related to recent fall versus metastasis.

Based on the history, physical examination, laboratory, and radiography investigation, the initial working diagnosis was esophageal carcinoma with SIADH. Management at that time was correction of hyponatremia with NaCl 3% based on the calculation of the patient's requirement. During treatment, the patient always complains of feelings of weakness, nausea but no vomiting, no appetite, headache, insomnia, and right chest pain that is temporarily improving by giving analgesics. There was no coughing and dyspnoea. Urine production per day was between 350 and 600 cc and irregularly defecating. On physical examination, blood pressure fluctuates within normal limits but once blood pressure reaches 160/100 mmHg, the patient has experienced a decrease in consciousness with laboratory results of sodium at that time: 108 mmol/L.

Laboratory results for four times electrolyte control tests after correction showed sodium levels of 108, 108, 122, and 112 mmol/L, respectively, never reached the normal levels although correction of sodium with 3% sodium based on the calculation of patient requirement was given repeatedly; low plasma osmolality and urine osmolality higher than the plasma osmolality were also found in this patient. Furthermore, the management of SIADH was given fluid restriction; hyponatremia was corrected with 3% sodium based on the calculation of the patient's requirement, injection of furosemide 40 mg intravenous daily, tetracycline 500 mg twice daily. We follow-up urine production daily, urine sodium, urine osmolality, and plasma osmolality.

The general condition of the patient improves; complaints often felt during hospitalization have diminished. Physical examination results: vital signs are within normal limits, and urine production increases around 1100 cc daily. Laboratory examination results: levels of sodium are 140 mmol/L. Results of urine examination are within normal limits (urine density: 1.010). The patient is permitted to outpatient with recommended continue oral therapy, calculate urine production daily, and electrolyte control.

## 3. Discussion

Syndrome of inappropriate antidiuretic hormone is a condition in which there is an increase of ADH release so that there is an increase in fluid reabsorption in the kidney which subsequently results in hyponatremia (dilutional hyponatremia). Under normal conditions, ADH or arginine vasopressin (AVP) is secreted from the posterior lobe of the pituitary gland in response to increased plasma osmolality or a decrease in plasma volume. In SIADH, increased ADH secretion does not depend on changes in plasma osmolality or volume [2]. The three main causes of SIADH are as follows: increased production of endogenous ADH (increased ADH secretion in posterior pituitary); increased production of exogenous ADH and idiopathic [1, 8–10].

SIADH is a form of paraneoplastic syndrome. The majority of SIADH in malignancies have been reported in small cell lung cancer patients, very rarely reported in patients with squamous cell esophageal carcinoma [3, 5, 10]. Certain types of cancer cells produce and secrete their own ADH, where the production of ADH occurs without regard to the needs of the body and then the kidneys will retain fluid, even when the body has excess fluid. Of all the cancers that produce their own ADH, small cell lung cancer is the most common cause of SIADH (75%) caused directly by the tumor [3]. The mechanism of the SIADH occurrence in small cell lung cancer is a result of the production of ectopic ADH by cancer cells and has also been shown to have aquaporins (AVP-regulated water channels) in the patient's kidneys. Recent studies report the expression of the vasopressin-neurophysin II (AVP-NP II) gene that controls AVP production in a small cell type of carcinoma cells [11, 12].

There is no theory that explains the mechanism of the SIADH occurrence in squamous cell carcinoma that is widely accepted. In general, studies on squamous cell carcinoma have found no AVP gene, but several neuropeptides associated with AVP have been detected [4, 12]. Physiologically, AVP synthesis in the hypothalamus begins with stimulation of several neuropeptides, including acetylcholine, histamine, bradykinin, angiotensin II, and neuropeptide Y [12]. In 2004, Lee et al. reported a significant increase in neuropeptide Y in the hypothalamus of experimental animals inoculated with human squamous cell carcinoma. The neuropeptide Y is produced by squamous cell carcinoma which stimulates endogenous AVP production. This mechanism is very different from the mechanism that occurs in small cell lung cancer where cancer cells directly produce ectopic AVP [13].

There are other causes of SIADH in cancer patients in addition to the direct result of tumor cells; some chemotherapy drugs used in malignant patients including vincristine, vinblastine, cisplatin, cyclophosphamide, and melphalan can also be a cause. Primary or metastatic tumors in the brain can also cause SIADH due to an increase in intracranial pressure [2, 3, 9].

Based on the modified Bartter and Schwartz criteria, the diagnosis of SIADH is established based on its existence: hyponatremia, decreased plasma osmolality (<275 mOsm/kg), increased urine osmolality (>100 mOsm/kg), euvolemic/hypervolemic, increased urinary Na^+^ excretion (>40 mmol/L), normal thyroid and adrenal function, and no use diuretic [3, 8, 9], and there are no abnormalities of the heart, kidneys, and liver [14].

This patient was diagnosed with squamous cell esophageal carcinoma and had received chemotherapy and radiotherapy 5 months before current hospital admission. In the course of the disease, the patient experiences persistent hyponatremia where sodium levels never reach normal levels, only fluctuating between 111 and 122 mmol/L even though they have been corrected by hypertonic 3% sodium based on the calculation of the patient's requirement. Clinical symptoms that appear at that time are in accordance with the symptoms that arise in moderate to severe hyponatremia which is feeling nauseous, loss of appetite, fatigue, and headache, even patients have experienced a decrease in consciousness. The patient was diagnosed with SIADH based on hyponatremia, low plasma osmolality based on the results of the calculation, patients without hypovolemia and no edema, no renal insufficiency, no thyroid abnormality, and no temporarily using diuretics.

Symptoms that arise in SIADH depend on the severity of the degree of hyponatremia and the rate at which hyponatremia occurs. The normal serum sodium concentration is 135-145 mmol/L. The patient may feel nausea, vomiting, decreased appetite, fatigue, headache, or without symptoms if the sodium concentration is at the level of 125-130 mmol/L. Symptoms of dizziness, hallucinations, and other neurological symptoms appear when sodium levels are between 115 and 125 mmol/L. Patients may experience more severe neurological symptoms such as seizures, coma, or death at a sodium concentration of less than 115 mmol/L. If sodium levels decrease very slowly, the patient can be asymptomatic even with a value of 110 mmol/L [4, 14].

There are three possibilities that are thought of as causes of SIADH in this patient: direct result of cancer cells, due to chemotherapy drugs that have been obtained, or due to the presence of metastasis to the brain which causes an increase in intracranial pressure. PET-CT results cannot confirm whether there is metastasis to the brain. Chemotherapy drugs as a cause have also not been eliminated because there is no data on the chemotherapy regimen that has been used. It was reported that some chemotherapy drugs for squamous cell cancer can cause SIADH [3, 5].

The current choice of SIADH therapy is that in addition to treating the underlying disease (if possible) and fluid restriction (first-line treatment for hyponatremia due to SIADH). A commonly recommended fluid restriction is around 800-1200 mL/day, adjusted for the severity of hyponatremia. Demeclocycline which is a tetracycline derivative is used in SIADH treatment because it causes nephrogenic diabetes insipidus in about 60% of patients. The mechanism of this drug is not yet known, and vasopressin resistance cannot be predicted. The onset of the drug is also unpredictable but usually 2-5 days after administration or can be even longer. Some health centers use lithium for SIADH therapy. Lithium also causes nephrogenic diabetes insipidus in about 30% of patients through its inhibition of vasopressin-stimulated aquaporin-2. The effect of this drug is unpredictable, and because of its side effects, not many doctors use it [15].

Furosemide is effective for rapid correction of hyponatremia in SIADH, but in long-term therapy, the efficacy is limited because diuresis also induces the occurrence of natriuresis so that it can worsen hyponatremia [15]. Because most SIADH cases have an increase in plasma vasopressin, the proposed treatment option is the administration of vasopressin-2 antagonist receptors, which specifically prevent reabsorption of fluid in renal tubules without effecting excretion of solute. This drug is called an aquaretic, a novel class of drugs that promote aquaresis [15]. Tolvaptan, an oral selective ADH receptor antagonist, may be an effective strategy treatment for hyponatremia secondary to SIAD, including noncancer medical patients. Similar results were described in the oncology setting. In chronic SIAD, the American guidelines indicate tolvaptan only if fluid restriction has failed and underline the lack of convincing data about the use of vaptans in patients with serum natrium values < 120 mmol/L [16].

There is data supporting that plasma sodium concentration can be increased in SIADH patients who are given normal saline (physiological saline), but therapy with normal saline is primarily intended only for patients who are difficult to distinguish whether hypovolemia or euvolemic hyponatremia; in this condition, the administration of normal saline infusion is safer as an initial measure of fluid restriction. Infusion with hypertonic saline (3% or 5%) is mainly given to patients with severe hyponatremia or patients at risk for neurological disorders. Treatments must be monitored closely given the risk of central pontine (extra pontine) myelinolysis in rapid administration [15]. In chronic hyponatremia, hypertonic saline is recommended for less than 10-12 mmol/L in the first 24 hours and can be given up to 18 mmol/L after 48 hours [17, 18].

The management that we did in this patient was fluid restriction of 1000 cc/day, infusion of 3% NaCl according to the calculation of patient need, injection of 20 mg furosemide daily, and tetracycline 500 mg twice daily, where treatment showed good results, as evidenced by reduced complaints, symptoms, and an increase in serum sodium and plasma osmolality.

The prognosis of SIADH depends on the cause and rate of hyponatremia. The mortality of patients with serum sodium levels less than 120 mmol/L is 25%, while serum sodium more than 120 mmol/L is 12.5%. Mortality in acute hyponatremia reaches 5-50% [7].

## 4. Summary

This case reports a 74-year-old male patient, who was diagnosed with squamous cell esophageal carcinoma with SIADH based on persistent hyponatremia and decreased plasma osmolality; he was not consuming diuretics, and no other background disease was suspected to cause SIADH except squamous cell esophageal carcinoma. Although malignancy is the most common cause of SIADH, it is very rare in squamous cell esophageal carcinoma. The symptoms of SIADH in these patients improve clinically and laboratory after treatment that consist of fluid restriction, administration of hypertonic sodium infusion, diuretic (furosemide), and tetracycline.

## Data Availability

No data were used to support this manuscript.
